# Red-Light Irradiation of Horse Spermatozoa Increases Mitochondrial Activity and Motility through Changes in the Motile Sperm Subpopulation Structure

**DOI:** 10.3390/biology9090254

**Published:** 2020-08-29

**Authors:** Jaime Catalán, Marion Papas, Sabrina Gacem, Yentel Mateo-Otero, Joan E. Rodríguez-Gil, Jordi Miró, Marc Yeste

**Affiliations:** 1 Equine Reproduction Service, Department of Animal Medicine and Surgery, Faculty of Veterinary Sciences, Autonomous University of Barcelona, ES-08193 Bellaterra (Cerdanyola del Vallès), Spain; dr.jcatalan@gmail.com (J.C.); papas.marion@gmail.com (M.P.); swp.sabrina.gacem@gmail.com (S.G.); juanenrique.rodriguez@uab.cat (J.E.R.-G.); 2 Biotechnology of Animal and Human Reproduction (TechnoSperm), Institute of Food and Agricultural Technology, University of Girona, ES-17003 Girona, Spain; yentel.mateo@udg.edu; 3 Unit of Cell Biology, Department of Biology, Faculty of Sciences, University of Girona, ES-17003 Girona, Spain

**Keywords:** photobiology, red-light, horse, semen, motility, mitochondrial activity

## Abstract

Previous studies in other mammalian species have shown that stimulation of semen with red-light increases sperm motility, mitochondrial activity, and fertilizing capacity. This study sought to determine whether red-light stimulation using a light emitting diode (LED) at 620–630 nm affects sperm motility and structure of motile subpopulations, sperm viability, mitochondrial activity, intracellular ATP levels, rate of O_2_ consumption and DNA integrity of horse spermatozoa. For this purpose, nine ejaculates were collected from nine different adult stallions. Upon collection, semen was diluted in Kenney extender, analyzed, its concentration was adjusted, and finally it was stimulated with red-light. In all cases, semen was packaged in 0.5-mL transparent straws, which were randomly divided into controls and 19 light-stimulation treatments; 6 consisted of a single exposure to red-light, and the other 13 involved irradiation with intervals of irradiation and darkness (light-dark-light). After irradiation, sperm motility was assessed using a Computerized Semen Analysis System (CASA). Flow cytometry was used to evaluate sperm viability, mitochondrial membrane potential and DNA fragmentation. Intracellular levels of ATP and O_2_ consumption rate were also determined. Specific red-light patterns were found to modify kinetics parameters (patterns: 4, 2-2-2, 3-3-3, 4-4-4, 5-1-5, and 5-5-5 min), the structure of motile sperm subpopulations (patterns: 2, 2-2-2, 3-3-3, and 4-1-4 min), mitochondrial membrane potential (patterns: 4, 3-3-3, 4-4-4, 5-1-5, 5-5-5, 15-5-15, and 15-15-15 min), intracellular ATP levels and the rate of O_2_ consumption (pattern: 4 min), without affecting sperm viability or DNA integrity. Since the increase in some kinematic parameters was concomitant with that of mitochondrial activity, intracellular ATP levels and O_2_ consumption rate, we suggest that the positive effect of light-irradiation on sperm motility is related to its impact upon mitochondrial activity. In conclusion, this study shows that red LED light stimulates motility and mitochondrial activity of horse sperm. Additional research is needed to address the impact of red-light irradiation on fertilizing ability and the mechanisms through which light exerts its effects.

## 1. Introduction

Artificial insemination (AI) is an effective technique to improve the use of stallions in breeding programs [1], and it is used worldwide not only for the reproduction of horses, but also for that of farm animals, companion animals, and wild species [2]. Its advantages are so vast that it is considered as a biotechnology with great impact on equine reproduction; in effect, when used correctly, a stallion can leave hundreds of descendants throughout its reproductive life [3]. While pregnancy rates following AI with any semen source have increased enough to provide commercially acceptable margins of reliability in the horse [4], equine veterinarians often find owners seeking methods that can improve the reproductive performance of their stallions. In this context, different strategies have been developed to improve and handle sperm, thus maximizing their survival and fertilizing ability [5,6].

Studies conducted with pig semen have reported that reproductive performance can be increased through light-stimulation of semen prior to AI [7,8]. In effect, previous works using different light sources, such as lasers and light-emitting diodes (LED), have suggested that light-stimulation has a positive effect on the motility and ability of sperm to fertilize the oocyte in pigs [7,8,9], mice [10], and sheep [11]. Remarkably, no study has reported any detrimental effect of visible light on DNA integrity [12,13,14]. Light-stimulation of semen consists of irradiating sperm samples at a fixed (laser) or variable (LED) wavelength for a short period of time [15]. Data from previous studies indicate that the effects of light-stimulation depend on the state of the sample, the irradiation of the light beam used [13], the time or pattern of exposure [7], and the species [11].

While the mechanisms through which light exerts its effects on sperm are largely unknown, mounting evidence gives endogenous cellular photosensitizers, especially those present in mitochondria, a crucial role. Consequently, when these molecules are stimulated with light, both the production of ATP and the entry of Ca^2+^ into the sperm cell raise [14]. This raise in energy supply underlies the subsequent increase of sperm motility [14,16,17,18]. In addition to this, opsins, which are present in sperm, and the potential influence of light on the conformation of other proteins like those belonging to the Transient Receptor Potential (TRP) family may also be involved in the sperm response to light [14,19]. 

The evidence accumulated so far in studies conducted with both low-level laser therapy devices and LED agree with these hypotheses, since they indicate that light irradiation accelerates mitochondrial respiration and ATP production in sperm [14,20,21]. Furthermore, it has become apparent that the sperm response to light-stimulation is biphasic, as low doses produce stimulating effects, moderate doses have no effect, and high doses exert cytotoxic effects [14].

As far as light devices are concerned, the positive results of low-level lasers and LED suggest that any method can be used safely [13]; however, practically speaking, not only are LED-based systems much cheaper and easier to maintain than lasers, but they also show high photonic efficiency [7]. Among all visible light spectra tested, red-light has been shown to be the one that improves sperm motility the most [15]. In addition to this, it has been reported that red-light stimulation (620–630 nm) by LED increases the fertilizing ability of pig sperm [7,8,9], without affecting their viability [7,9].

In spite of all the aforementioned, the effects of red-light stimulation on freshly ejaculated horse sperm are yet to be investigated. Therefore, the present work aimed at evaluating, for the first time, whether stimulation with red LED light (620–630 nm) affects the viability, motility, mitochondrial activity, intracellular levels of ATP, O_2_ consumption rate, DNA integrity, and motile sperm subpopulations of fresh horse spermatozoa.

## 2. Materials and Methods

### 2.1. Suppliers

All reagents used were of analytical grade and were purchased from Boehringer-Mannheim (Mannheim, Germany), Merck (Darmstadt, Germany), and Sigma-Aldrich (Saint Louis, MO, USA). As far as fluorochromes are concerned, and unless otherwise stated, all were purchased from Molecular Probes (Thermo Fisher Scientific; Waltham, MA, USA) and were previously resuspended with dimethyl sulfoxide (Sigma-Aldrich). Plastic materials were provided by Nunc (Roskilde, Denmark).

### 2.2. Animals and Ejaculates

This study included nine ejaculates from nine different adult stallions, with proven fertility, housed at the Equine Reproduction Service, Autonomous University of Barcelona (Bellaterra, Cerdanyola del Vallès, Spain). This is an EU-approved semen collection center (Authorization code: ES09RS01E) that operates under strict protocols of animal welfare and health control. All animals were semen donors and were collected under the health conditions set by the EU (free of equine arteritis, infectious anemia, and contagious metritis). Since this service already runs under the approval of the Regional Government of Catalonia (Spain) and because no manipulation to animals other than semen collection was carried out, the ethics committee of our institution indicated that no further ethical approval was required.

Ejaculates were collected through a Hannover artificial vagina (Minitüb GmbH, Tiefenbach, Germany) and an in-line nylon mesh filter was used to separate the gel fraction from the semen. Upon collection, gel-free semen was diluted 1:5 (v:v) with Kenney extender [22], previously prewarmed to 38 °C. Sperm concentration was determined in all samples through a hemocytometer (Neubauer Chamber, Paul Marienfeld GmbH & Co. KG, Lauda-Königshofen, Germany). 

### 2.3. Experimental Design

Prior to light-stimulation, sperm concentration of samples was adjusted to 25 × 10^6^ spermatozoa/mL. Following this, samples were packed into 0.5-mL transparent straws (Minitüb GmbH; Tiefenbach, Germany). After semen packaging, straws were placed in a programmable photo-activation system (Maxicow, IUL, S.A.; Barcelona, Spain). In this system, each straw is in contact with a triple LED configuration system that emits red-light (wavelength window: 620–630 nm). The system is equipped with a supporting software (IUL, S.A.) that allows the regulation of intensity and exposure time. In all cases, intensity was set at 100%.

A total of 19 irradiation protocols (red light) were evaluated. This aimed at determining which light exposure protocols had greater effects, which ones had no effect and which ones caused deleterious effects on sperm motility, viability, mitochondrial activity, intracellular levels of ATP, O_2_ consumption rate, or DNA integrity. Six of these protocols consisted of single periods of light emission (1, 2, 3, 4, 5, and 10 min), whereas the other 13 treatments consisted of light-dark-light intervals (1-1-1, 2-1-2, 2-2-2, 3-1-3, 3-3-3, 4-1-4, 4-4-4, 5-1-5, 5-5-5, 10-5-10, 10-10-10, 15-5-15, and 15-15-15 min). Control samples were not irradiated. In order to determine the impact of each irradiation pattern, sperm viability, motility and motile subpopulations, mitochondrial activity, intracellular levels of ATP, O_2_ consumption rate, and DNA fragmentation were assessed. Prior to evaluation, irradiated and control samples, previously transferred into 1.5-mL tubes, were incubated in a water bath at 38 °C for 5 min.

### 2.4. Analysis of Sperm Motility

Sperm motility was evaluated using a computer-assisted sperm-analysis (CASA) system (Integrated Sperm Analysis System V1.0; Proiser S.L.; Valencia, Spain) following incubation at 38 °C for 5 min. Five μL of each sperm sample was placed onto a Makler chamber (Sefi Medical Instruments; Haifa, Israel) previously warmed at 38 °C. Samples were analyzed with a 10 × negative phase-contrast objective and an Olympus BX41 microscope (Olympus), and at least 1000 sperm cells per analysis were counted. In each evaluation, percentages of total (%TMOT) and progressively motile spermatozoa (%PMOT) were recorded together with the following kinetic parameters: curvilinear velocity (VCL, μm/s), which is the mean path velocity of the sperm head along its actual trajectory; straight-line velocity (VSL, μm/s), which is the mean path velocity of the sperm head along a straight line from its first to its last position; average path velocity (VAP, μm/s), which is the mean velocity of the sperm head along its average trajectory; percentage of linearity (LIN, %), which is the quotient between VSL and VCL multiplied by 100; percentage of straightness (STR, %), which is the quotient between VSL and VAP multiplied by 100; percentage of oscillation (WOB, %), which is the quotient between VAP and VCL multiplied by 100; mean amplitude of lateral head displacement (ALH, μm), which is the mean value of the extreme side-to-side movement of the sperm head in each beat cycle; and frequency of head displacement (BCF, Hz), which is the frequency at which the actual sperm trajectory crosses the average path trajectory (Hz). In addition, individual kinetic parameters for each spermatozoon were also recorded and used to investigate the effects of light-stimulation upon sperm motile subpopulations.

Settings of the CASA system were those recommended by the manufacturer, i.e., frames/s: 25 images captured per second; particle area >4 and <75 µm^2^; connectivity: 6; minimum number of images to calculate the ALH: 10. Cut-off values were VAP ≥ 10 μm/s for a sperm cell to be considered as motile, and STR ≥ 75% for being considered as progressively motile.

### 2.5. Flow Cytometry Analyses

#### 2.5.1. General Information about Flow Cytometry Analyses

Information about flow cytometry analyses is given according to the recommendations of the International Society for Advancement of Cytometry [23]. Prior to staining, sperm concentration (except for SCSA test) was adjusted to 1 × 10^6^ total spermatozoa per mL in a final volume of 0.5 mL with HEPES buffered saline solution (10 mM HEPES, 150 mM NaCl, 10% BSA; pH = 7.4). In addition, a correction procedure that consisted of differentiating into DNA-containing and non-DNA-containing particles was made for JC1 test, since the presence of alien particles could overestimate the percentages of intact spermatozoa [24,25].

All flow cytometry assessments were conducted using a Cell Laboratory QuantaSC cytometer (Beckman Coulter; Fullerton, CA, USA), and samples were excited with an argon ion laser (488 nm) set at a power of 22 mW. For each event, the cytometer provided the electronic volume (EV, equivalent to forward scatter, FS, in other equipment) and the side scatter (SS). Calibration of this device was made periodically through 10-µm Flow-Check fluorospheres (beads; Beckman Coulter); the bead size was positioned at channel 200 on the volume scale.

A total of three optical filters (FL1, FL2 and FL3), with the following particular characteristics, were used: FL1 (green fluorescence): Dichroic/Splitter, DRLP: 550 nm, BP filter: 525 nm; FL2 (orange fluorescence): DRLP: 600 nm, BP filter: 575 nm; and FL3 (red fluorescence): LP filter: 670/730 nm. Signals were logarithmically amplified and photomultiplier settings were adjusted to particular staining methods. FL1 was used to detect green fluorescence (SYBR14, JC1 monomers and SCSA), FL2 was used to detect orange fluorescence (JC1 aggregates, JC1_agg_), and FL3 was used to detect red fluorescence (PI and SCSA). When required, and as stated below, compensation was used to minimize spill over between channels.

Sheath flow rate was set at 4.17 µL/min in all analyses, and EV and SS were recorded in a log-linear mode (in EV vs. SS dot plots) for a minimum of 10,000 events per assessment. The analyzer threshold was adjusted on the EV channel to exclude subcellular debris and cell aggregates, and the sperm-specific events were positively gated on the basis of EV/SS distributions. Information on the events was collected in list-mode data files (*.LMD), and files were subsequently analyzed through the Cell Lab QuantaSC MPL Analysis Software (version 1.0; Beckman Coulter). Three replicates using independent tubes were evaluated, and the corresponding mean ± standard error of the mean (SEM) was subsequently calculated.

#### 2.5.2. Plasma Membrane Integrity

Sperm membrane integrity was assessed using the LIVE/DEAD Sperm Viability Kit (SYBR14/PI; Molecular Probes, ThermoFisher Scientific, Waltham, Massachusetts, MA, USA), according to the protocol described by Garner and Johnson [26] adapted to horse spermatozoa. Briefly, sperm samples were incubated at 37.5 °C for 10 min with SYBR14 at a final concentration of 100 nM, and then with PI at a final concentration of 12 µM for 5 min at the same temperature. All incubations were performed in the dark. FL1 was used to measure the green fluorescence from SYBR14, and FL3 was used to detect the red fluorescence from PI. Three sperm populations were identified: (i) spermatozoa with an intact plasma membrane, stained in green (SYBR14^+^/PI^−^); (ii) spermatozoa with a damaged plasma membrane, stained in red (SYBR14^−^/PI^+^); and (iii) spermatozoa with a damaged plasma membrane, stained in orange (SYBR14^+^/PI^+^). Non-sperm particles (debris) were found in the SYBR14^−^/PI^−^ quadrant, and were used to correct JC1-data. Single-stained samples were used for setting the EV-gain, FL1 and FL3 photomultiplier (PMT)-voltages, and for compensation of SYBR14 spill over into the PI channel (2.45%).

#### 2.5.3. Evaluation of Mitochondrial Membrane Potential (∆Ψm, JC1)

Mitochondrial membrane potential of horse spermatozoa was determined after modification of the protocol described in [27]. Sperm samples were incubated with JC1 (5,5′,6,6′-tetrachloro-1,1′,3,3′tetraethyl-benzimidazolylcarbocyanine iodide) at a final concentration of 0.5 μM at 37.5 °C for 30 min in the dark. Green fluorescence from JC1-monomers was collected through FL1, and orange fluorescence from JC1 aggregates (JC1_agg_) was collected through FL2. Two populations were distinguished: (i) spermatozoa with low MMP, in which all mitochondria were stained in green (FL1^+^/FL2^−^); and (ii) spermatozoa with high MMP (JC1_agg_), which contained spermatozoa with heterogeneous mitochondria stained both in green and orange in the same cell (FL1^+^/FL2^+^) and spermatozoa that had all their mitochondria stained in orange (FL1^−^/FL2^+^). Spillover of FL1 into FL2-channel was compensated (68.5%). Following the protocol of Petrunkina et al. [24], the percentages of non-sperm, debris particles found in the SYBR14/PI test (SYBR14^−^/PI^−^) were used to correct the percentages of non-stained events in the sperm population with low MMP; the percentages of the sperm population with high MMP were recalculated.

#### 2.5.4. DNA Integrity (SCSA Test)

DNA fragmentation of horse spermatozoa in control and irradiated samples was evaluated through SCSA test [28,29], as modified by Morrell et al. [30]. Briefly, sperm samples were diluted in a buffer solution (TNE; 0.15 M NaCL, 0.01 M Tris-HCl, 1 mM EDTA, pH = 7.4) to a final concentration of 2 × 10^6^ spermatozoa/mL. Next, 200 µL of this solution containing 2 × 10^6^ spermatozoa/mL were mixed with 400 µL of an acid-detergent solution (80 mM HCl, 150 mM NaCl, and 0.1% Triton X-100; pH = 1.2) in ice. After 30 s, 1.2 mL of an acridine orange (AO) solution (6 μg/mL in 37 mM citric acid, 126 mM Na_2_HPO_4_, 1.1 mM EDTA, 150 mM NaCl, pH = 6.0) was added, and samples were kept in ice for further 3 min. Immediately after this time, samples were evaluated and green and red fluorescence were collected through FL1 and FL3 filters, respectively. Percentages of DNA fragmentation (DNA fragmentation index, %DFI), which was the ratio between red (ssDNA) fluorescence and red (ssDNA) + green (dsDNA) fluorescence, mean fluorescence intensity of single stranded DNA (ssDNA, mean DFI) and percentages of high DNA stainability (HDS) were determined.

### 2.6. Determination of Intracellular ATP Levels

Intracellular ATP levels were determined following the protocol set by Chida et al. [31]. Briefly, after irradiated, 1-mL semen aliquots were centrifuged at 17 °C for 30 s and pellets were immediately plunged into liquid N_2_. Frozen pellets were subsequently stored at −80 °C for three weeks. Thereafter, pellets were resuspended in 300 µL ice-cold 10 mM 2-[4-(2-hydroxyethyl) piperazin-1-yl]ethanesulfonic acid (HEPES) buffer containing 250 mM sucrose (pH was adjusted to 7.4). Resuspended pellets were sonicated (10 kHz, 20 pulses; Bandelin Sonopuls HD 2070; Bandelin Electronic GmbH and Co., Berlin, Germany), while being kept on ice to avoid specimen heating. Samples were subsequently centrifuged at 1000× g and 4 °C for 10 min and supernatants were collected. Twenty µL was used to determine total protein content, and the remaining volume was mixed with 300 µL ice-cold 10% (v:v) trichloroacetic acid and kept at 4 °C for 20 s. Samples were subsequently centrifuged at 1000× *g* and 4 °C for 30 s, and supernatants were carefully separated from the pellet and again centrifuged at 1000× *g* and 4 °C for 10 min. Resulting supernatants were mixed with two volumes of 1 M Tris-acetate buffer (pH = 7.75), and ATP content was determined in these final suspensions using the Invitrogen^®^ ATP Determination Kit (ThermoFisher Bioscientific; Waltham, MA, USA; catalogue number: A22066) following the manufacturer’s instructions. Determinations of ATP content were carried out through an Infinite F200 fluorimeter (TECAN^®^), using 96-wells microplates for fluorescence-based assays (Invitrogen^®^). To normalize data, total protein of samples was determined through the Bradford method [32] using a commercial kit (Bio-Rad laboratories; Hercules, CA, USA).

### 2.7. Determination of O_2_ Consumption Rate

For the determination of the O_2_ consumption rate, the unirradiated control sample and five light stimulation patterns of short and long exposure to light were used, which had obtained significant differences in some kinetic parameters of sperm motility and mitochondrial activity, these parameters were; 2, 4, 3-3-3, 5-5-5, and 15-5-15 min. Determination of O_2_ consumption rate was performed using the SensorDish^®^ Reader (SDR) system (PreSens Gmbh; Regensburg, Germany). One-mL semen aliquots, previously exposed to red-light, were transferred onto Oxodish^®^ OD24 plates (24 wells/plate) specifically designed for this device. Plates were sealed with Parafilm^®^, introduced in the SDR system, and incubated at 37 °C (controlled atmosphere) for 2 h. During that time, O_2_ concentration was recorded in each well at a rate of one reading per min. Data were exported to an Excel file and final O_2_ consumption rate was normalized against the total number of viable spermatozoa per sample, which was determined through flow cytometry (SYBR14^+^/PI^−^ spermatozoa) using another aliquot. In addition, O_2_ consumption rates in irradiated samples were standardized against the control (O_2_ consumption rate _irradiation pattern_/O_2_ consumption rate _control_ × 100).

### 2.8. Statistical Analyses

Statistical analyses were conducted using a statistical package (IBM^®^ SPSS^®^ 25.0 for Windows; IBM corp., Armonk, NY, USA). Data were first tested for normality and homogeneity of variances with Shapiro–Wilk and Levene tests, respectively. When required, data were transformed through arcsin √x. The effects of red-light stimulation patterns on total and progressive motility, kinematic parameters, percentages of viable spermatozoa, percentage of spermatozoa with high MMP, intracellular ATP levels, O_2_ consumption rates, and DNA fragmentation were evaluated through one-way analysis of variance (ANOVA) followed by post-hoc Sidak’s test.

Sperm motile subpopulations were set according to the procedure described in [33]. Briefly, individual kinematic parameters obtained for each spermatozoon (VSL, VCL, VAP, LIN, STR, WOB, ALH, and BCF) were used as independent variables in a principal component analysis (PCA). These kinematic parameters were sorted into PCA components and the obtained matrix was subsequently rotated using the Varimax procedure with the Kaiser normalization. For each spermatozoon, regression scores of the resulting PCA components were worked out.

Based on the regression scores of each individual spermatozoon, a two-step cluster analysis based on the log-likelihood distance and the Schwarz’s Bayesian Criterion was run. Following identification of four motile sperm subpopulations, percentages of spermatozoa belonging to each subpopulation (SP1, SP2, SP3, or SP4) were calculated in each treatment and replicate. These percentages were subsequently used to evaluate the effects of red-light stimulation on sperm subpopulations through one-way ANOVA and Sidak’s post-hoc test.

In all analyses, the level of s for statistical significance were set at *p* ≤ 0.05. Data are shown as mean ± standard error of the mean (SEM).

## 3. Results

### 3.1. Effects of Red-Light Irradiation on Sperm Viability

**Figure 1:**
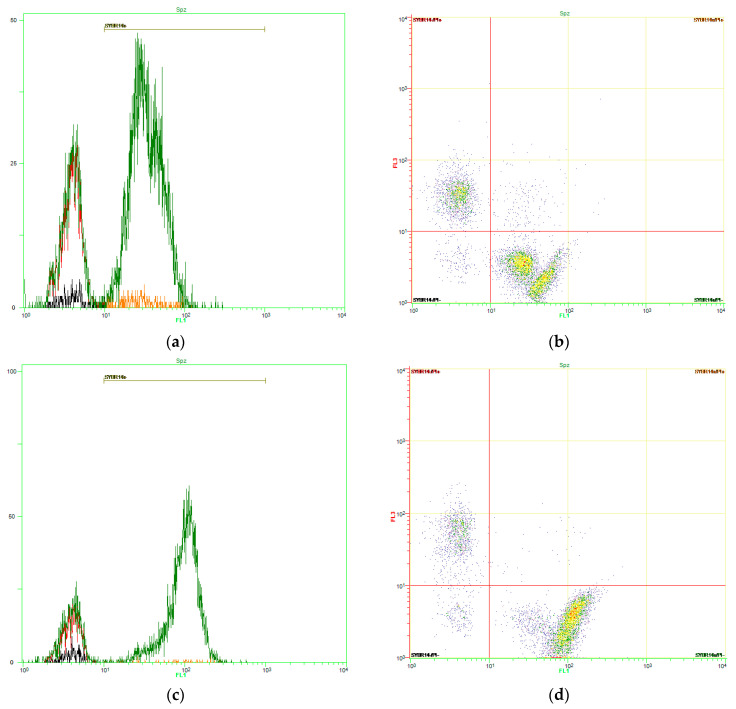
Sperm viability (SYBR14/PI). Representative SYBR14 (FL1) histograms for the control (**a**) and 15-15-15 light-stimulation pattern (**c**). Representative FL1/FL3 dot-plots for the control (**b**) and 15-15-15 light-stimulation pattern (**d**). Viable spermatozoa (SYBR14^+^/PI^−^) appear in the lower right quadrant. No significant differences between the control and treatments were observed (*p* > 0.05).

**Figure 2:**
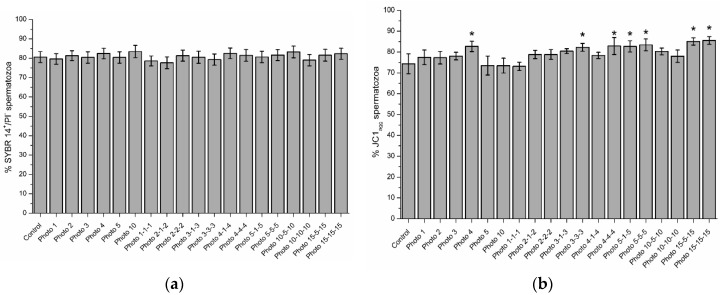
(**a**) Percentages of spermatozoa with an intact plasma membrane (SYBR14^+^/PI^−^; viable spermatozoa) in the control and the different light-stimulation patterns. No significant differences between the control and light-stimulation patterns were observed (*p* > 0.05). (**b**) Percentages of spermatozoa with high mitochondrial membrane potential (JC1_agg_) in the control and different irradiation patterns. The superscript (*) means significant differences (*p* ≤ 0.05) between the control and the different light-stimulation patterns. Data are shown as mean ± SEM of nine independent experiments.

Figure 1 shows representative histograms and dot-plots for the control (Figure 1a,b) and one light-stimulation pattern (15-15-15; Figure 1c,d). Figure 2a shows, as mean ± SEM, the percentages of viable spermatozoa following light-stimulation. No significant differences between control and irradiation patterns were observed (*p* > 0.05).

### 3.2. Effects of Red-Light Irradiation on Mitochondrial Membrane Potential (ΔΨm)

**Figure 3:**
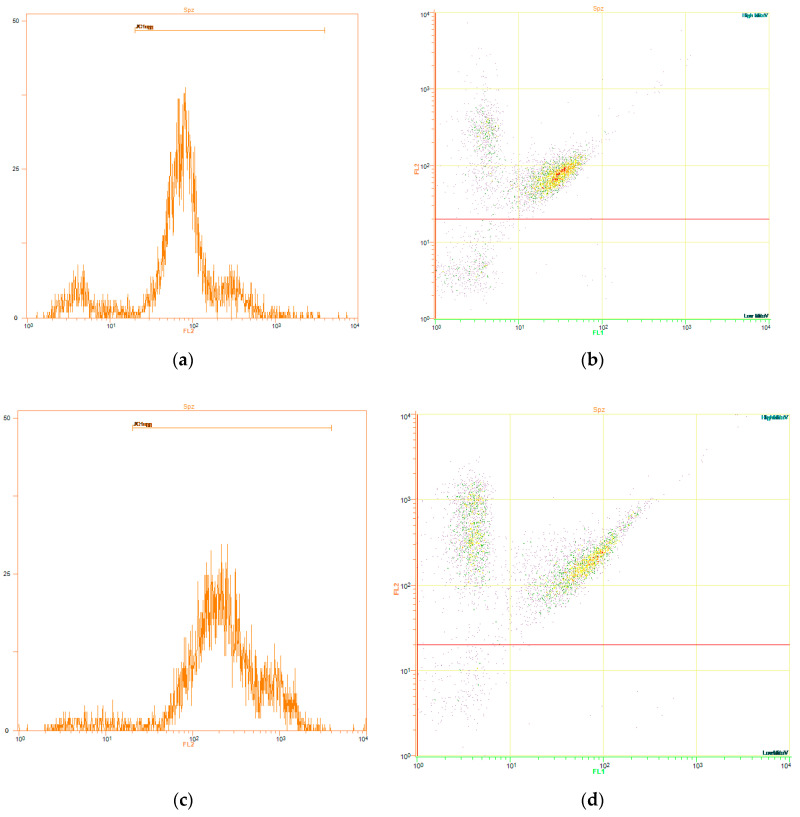
Mitochondrial membrane potential (ΔΨm, JC1). Representative JC1_agg_ (FL2 channel) histograms for the control (**a**) and 15-15-15 light-stimulation pattern (**c**). Representative FL1/FL2 dot-plots for the control (**b**) and 15-15-15 light-stimulation pattern (**d**). Spermatozoa with high mitochondrial membrane potential (JC1_agg_) appear in the upper half of the Figure. Percentages of spermatozoa with high mitochondrial membrane potential (JC1_agg_) were significantly (*p* < 0.05) higher in the 15-15-15 light-stimulation pattern than in the control.

As shown in Figure 2b, percentages of spermatozoa with high JC1_agg_ were significantly (*p* < 0.05) higher, compared with the control samples (74.4% ± 4.8%), in the following light-stimulation patterns: 4 (82.7% ± 2.5%), 3-3-3 (82.3% ± 1.9%), 4-4-4 (82.9% ± 4.1%), 5-1-5 (82.7% ± 2.7%), 5-5-5 (83.4% ± 2.8%), 15-5-15 (85.1% ± 1.7%), and 15-15-15 (85.5% ± 1.8%). Figure 3 shows representative histograms and dot-plots for the control (Figure 3a,b) and 15-15-15 pattern (Figure 3c,d).

### 3.3. Effects of Red-Light Irradiation on Sperm Motility

**Figure 4:**
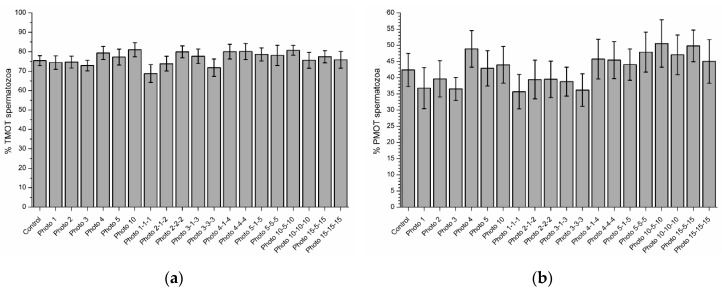
Percentages of total (**a**; TMOT) and progressively motile spermatozoa (**b**; PMOT) in the control and different irradiation patterns. No significant differences between the control and light-stimulation patterns were observed. Data are shown as mean ± SEM of nine independent experiments.

Total and progressive sperm motility did not differ between control and irradiation patterns (*p* > 0.05; Figure 4). However, red-light stimulation affected several sperm kinematic parameters when compared to the control in a fashion that depended on the specific light regime applied to cells. In effect, VSL increased after the application of irradiation patterns 3-3-3 (65.7 µm/s ± 4.8 µm/s) and 5-5-5 (66.7 µm/s ± 2.0 µm/s) when compared to control samples (56.9 µm/s ± 2.5 µm/s; see Table 1). Furthermore, VAP significantly (*p* < 0.05) increased with patterns 4 (94.4 µm/s ± 4.6 µm/s), 2-2-2 (96.0 µm/s ± 4.7 µm/s), 3-3-3 (96.4 µm/s ± 5.6 µm/s), and 4-4-4 (94.8 µm/s ± 3.9 µm/s) with regard to the control (81.9 µm/s ± 4.7 µm/s; see Table 1). Finally, BCF significantly (*p* < 0.05) decreased after the application of patterns 3-3-3 min (8.4 Hz ± 0.6 Hz) and 5-1-5 (8.4 Hz ± 0.6 Hz) when compared to the control (9.7 Hz ± 0.6 Hz; Table 1).

### 3.4. Sperm Subpopulations

Four different sperm subpopulations were identified following cluster analyses based on the analyzed individual kinematic parameters. Table 2 shows the kinematic parameters for these sperm subpopulations, which were identified as SP1, SP2, SP3, and SP4. SP1 was the slowest sperm subpopulation, since it showed low VCL, VSL, VAP, and ALH. SP2 presented intermediate values (higher than SP1 but lower than SP3) in most sperm kinetic parameters (VCL, VSL, VAP, LIN, STR, and WOB) and exhibited higher ALH than SP1 and SP3. In addition, SP2 also showed the highest BCF. SP3 was the most linear subpopulation, as displayed the highest values in most kinetic parameters (i.e., VSL, VAP, LIN, STR, and WOB). Finally, SP4 was the subpopulation that, despite showing the highest values of VCL and ALH, its VSL was similar to that of SP1, which was the slowest subpopulation (SP1), and its LIN, STR, and WOB was lower than in the other three subpopulations (SP1, SP2, and SP3).

**Figure 5:**
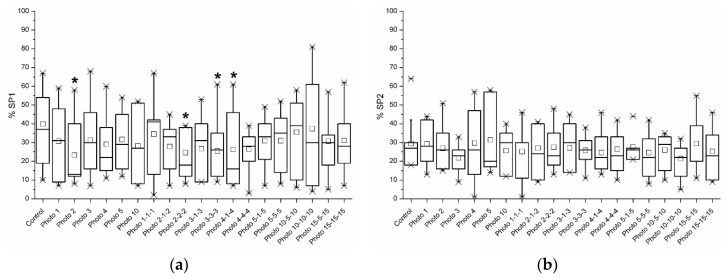

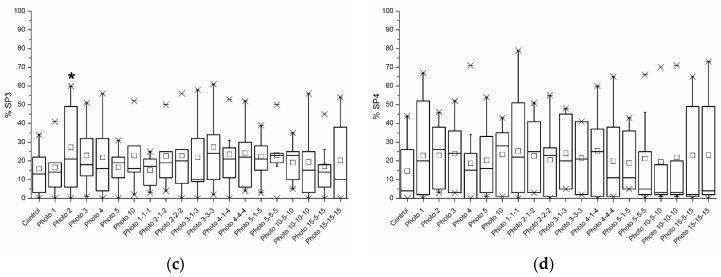
Box plots showing the percentages of sperm belonging to (**a**) Subpopulation 1 (SP1, which was the slowest subpopulation based on VCL, VSL, and VAP); (**b**) Subpopulation 2 (SP2, which presented intermediate values, higher than SP1 but lower than SP3); (**c**) Subpopulation 3 (SP3, which was the one with the highest VSL, VAP, and LIN); and (**d**) Subpopulation 4 (SP4, which was the one with the highest VCL and ALH but with the lowest LIN, STR, and WOB) in the control and different irradiation patterns. The superscript (*) means significant differences (*p* ≤ 0.05) between the control and irradiation patterns. Data are shown as mean ± SEM of nine independent experiments.

Figure 5a shows the percentages of spermatozoa belonging to SP1. These percentages were significantly (*p* < 0.05) higher in the control (39.8% ± 5.8%) than in the following irradiation patterns: 2 (23.3% ± 5.1%), 2-2-2 (24.6% ± 4.4%), 3-3-3 (25.5% ± 5.1%), and 4-1-4 (26.3% ± 5.8%). Percentages of spermatozoa belonging to SP3 significantly (*p* < 0.05) increased following light-stimulation for 2 min (27.2% ± 6.9% vs. 15.8% ± 3.4% in the control; Figure 5c). In contrast, no significant differences (*p* > 0.05) between the control and irradiation patterns were observed in SP2 and SP4 (Figure 5b,d).

### 3.5. Effects of Red-Light Stimulation on Intracellular ATP Levels

**Figure 6:**
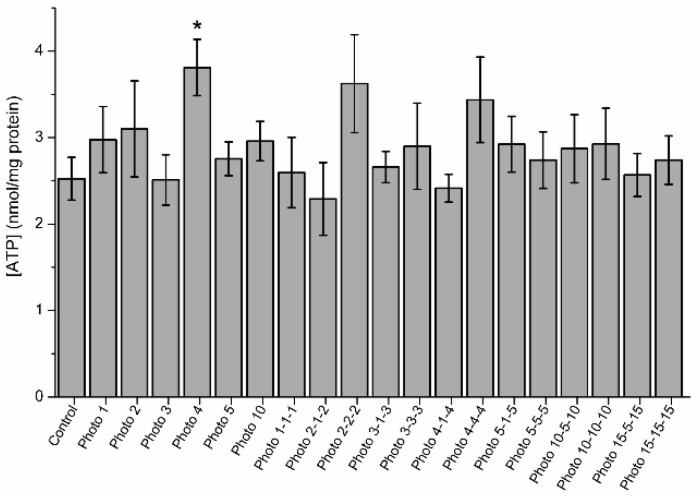
Intracellular ATP levels in control and irradiated samples. The superscript (*) means significant differences (*p* ≤ 0.05) between the control and different light-stimulation patterns. Data are shown as mean ± SEM of nine independent experiments.

Figure 6 shows intracellular ATP levels in irradiated and control samples. Samples irradiated for 4 min showed significantly (*p* < 0.05) higher intracellular ATP levels (3.8 nmol/mg protein ± 0.3 nmol/mg protein) than the control (2.5 nmol/mg protein ± 0.2 nmol/mg protein). No significant differences between the control and the other light-stimulation patterns were observed.

### 3.6. Effects of Red-Light Stimulation on Oxygen Consumption

**Figure 7:**
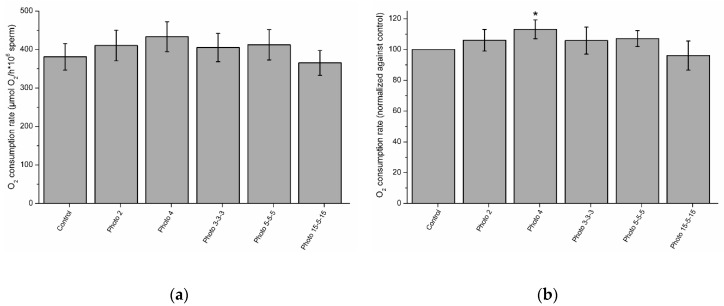
O_2_ consumption rates in control and irradiated samples (**a**)**,** and rates in irradiated samples normalized against the corresponding control (**b**). The superscript (*) means significant differences (*p* ≤ 0.05) between the control and irradiation patterns. Data are shown as mean ± SEM of nine independent experiments.

Figure 7a shows O_2_ consumption rates, which did not differ (*p* > 0.05) between irradiated and control samples. However, when, in order to remove individual variability, O_2_ consumption rates in irradiated samples were normalized against their corresponding controls (Figure 7b), those standardized rates in samples irradiated for 4 min were significantly (*p* < 0.05) higher than in the control.

### 3.7. Effects of Red-Light Stimulation on DNA Fragmentation

As shown in Table 3, percentages of DNA fragmentation (% DFI), mean fluorescence intensity of single-stranded DNA (mean DFI), and percentages of high DNA stainability (% HDS) did not differ (*p* > 0.05) between the control and irradiated samples.

## 4. Discussion

Our results clearly show that irradiation with LED-based red-light modifies some kinetic parameters and the structure of motile sperm subpopulations. These changes occurred together with an increase in mitochondrial activity of horse spermatozoa, without affecting the integrity of plasma membrane or DNA. Interestingly, our data suggest that the effects of exposing horse spermatozoa to red-light heavily rely upon the specific utilized light-dark interval. These findings are similar to those observed in previous studies based on the use of both laser and LED-based light sources in species such as dogs [17], buffalos [34], and humans [35].

In addition, our data are consistent with those reported by other authors, who have suggested that irradiation effects depend on the precise rhythm and intervals of light-darkness regardless of the light source (LED or laser) used [7,36,37]. In fact, distinct wavelengths have different effects and, although the optimal wavelength varies between species, mounting evidence indicates that blue- or green-light is detrimental for mammalian spermatozoa [11,13,38]. Related with this, it is important to emphasize that, in previous studies carried out in other mammalian species, red-light has been demonstrated to be the one that mostly improves motility and other functional parameters in both human and animal spermatozoa (see [14] for review).

The results shown herein suggest that stallion sperm exposed to short patterns of red-light stimulation, both single or combining light with dark regimens, exhibit better response on motility parameters than the long ones. This is very apparent when looking at the changes in kinetic parameters (patterns 4, 2-2-2, 3-3-3, 4-4-4, 5-1-5, and 5-5-5), and in the sperm subpopulation structure (patterns 2, 2-2-2, 3-3-3, and 4-1-4). In this context, it is worth mentioning that, in a study conducted using liquid-stored boar semen irradiated with LED red-light, the shortest irradiation patterns were the ones that exerted the most intense effects [7]. A similar result was also observed in a study conducted with human sperm, in which irradiation with a single exposition to red LED light for 50 s, 100 s, and 200 s increased sperm motility, whereas the exposure for 400 s had inhibitory effects [13]. Therefore, all these results, including those of our study, would be consistent with the fact that the two-phase response to light-doses follows the Arndt–Schultz curve [38,39]. In fact, these data also suggest that low levels of irradiation have greater but variable effect on tissues than higher levels of irradiation. This variable response, including the impact upon sperm motility, has already been described elsewhere [12,13,35,37,40,41] although one study with human sperm suggested a linear relationship between the dose of red-light irradiation and sperm motility [42]. However, taking into account the considerable difference in the response to light between species, a more in-depth study is needed to establish better the mechanisms underlying the observed variability.

Focusing on sperm motility, our findings in some kinetic patterns were similar to those observed in other species. These parameters were VSL (patterns: 3-3-3 min and 5-5-5 min), with similar effects to those found in humans [41], dogs [17], buffalos [34], and pigs [7]; and VAP (patterns: 4 min, 2-2-2 min, 3-3-3 min, and 4-4-4 min), with a similar impact to that reported in dogs [16,17], bulls [43], buffalos [34], and pigs [7]. In addition, we observed a significant decrease in BCF (patterns 3-3-3 min and 5-1-5 min) compared to the control, which matched with previous reports in humans [42], but differed from what found in dogs [16,17] and pigs [7].

Another important point to highlight is the lack of differences in total and progressive motility between control and light-stimulated samples. While these results are in agreement with those previously reported in dogs [16,17] and cattle [43], irradiation of sperm with red-light has been found to increase total and progressive motility in humans [35,37,41,42,44], buffalos [34], sheep [45], and pigs [7]. Therefore, the aforementioned differences also support that the effects of light-dose on sperm function rely upon species [11].

When evaluating the presence of sperm motile subpopulations in horse ejaculates, we identified four separate subpopulations. These results are similar to those reported previously in horses [46], donkeys [47], cattle [48], and goats [49,50]. Remarkably, we observed that percentages of spermatozoa belonging to SP1, which was the slowest subpopulation based on VCL, VSL, and VAP, were significantly higher in the control than after the following light-stimulation patterns: 2 min, 2-2-2 min, 3-3-3 min, and 4-1-4 min. In addition, percentages of sperm belonging to SP3, which showed the highest values in most kinetic parameters, including VSL, VAP, and LIN, were significantly lower in the control than following light-stimulation for 2 min. Therefore, our data suggest that irradiation of horse sperm with red-light modifies the structure of motile sperm subpopulations by decreasing the percentage of the slowest sperm subpopulation and increasing the percentage of the most linear and fastest one. It is worth mentioning that these results are in agreement with a previous study conducted with dog semen, in which stimulation with laser red-light significantly increased the proportions of the fastest sperm subpopulation [17]. These changes in the characteristics of motile sperm subpopulations, in addition to those observed in sperm kinetic parameters, indicate that not only does irradiation with red-light increase sperm velocity, but it also modifies the way through which sperm move. At this moment, there is no clear explanation of how these effects occur, since the exact mechanism(s) through which red-light stimulates sperm remain(s) unclear. However, it has been hypothesized that a mechanism related to the activation of sperm mitochondria could be essential to explain those effects [14]. In fact, our results from the analysis of mitochondrial membrane potential agree with this possibility, since irradiation with patterns 4, 3-3-3, 4-4-4, 5-1-5, 5-5-5, 15-5-15, and 15-15-15 increased the percentages of spermatozoa with high ΔΨm. A similar increase on ΔΨm has been described in pig sperm [7,43]. Thus, these data suggest that stimulation with red-light could increase mitochondrial activity through photosensitizers that are present in the electronic chain, such as cytochrome C [7,51], which would result in higher sperm motility and greater fertilization potential [52].

Recent studies have concurred that oxygen consumption is an alternative way to measure mitochondrial activity, which could be better than the use of markers of mitochondrial membrane potential, such as JC1 [53,54]. In addition to this, the rate of oxygen consumption also provides an indirect measure of ATP produced by oxidative phosphorylation in sperm [54]. The results obtained in this study agree with this possibility, since they showed an increase in intracellular ATP levels and O_2_ consumption rate (O_2_ consumption rate normalized against the corresponding control) in samples irradiated for 4 min, compared with the non-irradiated control. Furthermore, these results agree with those obtained in the evaluation of the percentages of viable sperm with high MMP with this stimulation pattern. In this context, it is important to take into account that the increase in the potential of the mitochondrial membrane is associated with changes in the consumption of ATP and the activity of the enzymes of the respiratory chain [55,56]. Related with this, Iaffaldano et al. [45] observed that light stimulation of frozen-thawed ram sperm with a He-Ne laser increased ATP content and the activity and affinity of cytochrome C oxidase (CCO) for their substrate (cytochrome C). Interestingly, these authors found that CCO activity and ATP content were positively correlated with each other and with sperm motility, supporting the hypothesis that the effects of red light upon sperm are mediated by mitochondria.

## 5. Conclusions

In conclusion, this work has shown, for this first time, that irradiation of horse sperm with red-light modifies the structure of motile sperm subpopulations, increases some kinetic parameters, intracellular ATP levels, the rate of O_2_ consumption, and mitochondrial membrane potential, without affecting the integrities of DNA and plasma membrane. Therefore, we suggest that the effects of light on sperm are related to mitochondrial function. These effects, however, rely upon the specific light-stimulation pattern. While these changes could have a beneficial impact upon the fertility ability of horse spermatozoa, further research investigating whether such a positive effect exists is much warranted. In addition to contemplating in vivo or in vitro fertility assays, future studies should also address which mechanism(s) underlie(s) this sperm response to red light.

## Figures and Tables

**Table 1:** Kinetic parameters (mean ± SEM) of horse spermatozoa in the control and different irradiation patterns.

Patterns	VCL (µm/s)	VSL (µm/s)	VAP (µm/s)	LIN (%)	STR (%)	WOB (%)	ALH (µm)	BCF (Hz)
Control	107.2 ± 5.9 ^a^	56.9 ± 2.5 ^a^	81.9 ± 4.7 ^a^	55.6 ± 4.7 ^a^	71.0 ± 4.6 ^a^	77.6 ± 2.1 ^a^	2.7 ± 0.1 ^a^	9.7 ± 0.6 ^a^
Ph 1	116.0 ± 8.3 ^a^	58.9 ± 3.9 ^a^	88.5 ± 4.7 ^a^	52.2 ± 6.2 ^a^	66.3 ± 5.7 ^a^	77.2 ± 2.8 ^a^	2.9 ± 0.2 ^a^	9.3 ± 0.4 ^a^
Ph 2	117.2 ± 4.8 ^a^	62.3 ± 5.3 ^a^	92.0 ± 4.9 ^a^	53.6 ± 4.8 ^a^	68.0 ± 4.2 ^a^	78.1 ± 2.6 ^a^	2.9 ± 0.1 ^a^	9.1 ± 0.3 ^a^
Ph 3	113.2 ± 7.0 ^a^	60.7 ± 4.0 ^a^	90.4 ± 6.3 ^a^	54.5 ± 4.2 ^a^	68.2 ± 4.5 ^a^	79.6 ± 2.1 ^a^	2.9 ± 0.2 ^a^	9.1 ± 0.2 ^a^
Ph 4	117.7 ± 5.2 ^a^	62.5 ± 4.5 ^a^	94.4 ± 4.6 ^b^	53.2 ± 4.6 ^a^	66.2 ± 4.8 ^a^	80.2 ± 2.2 ^a^	3.0 ± 0.3 ^a^	9.2 ± 0.8 ^a^
Ph 5	111.3 ± 6.6 ^a^	58.9 ± 2.6 ^a^	87.7 ± 4.3 ^a^	54.8 ± 5.0 ^a^	68.6 ± 5.0 ^a^	79.2 ± 2.0 ^a^	2.6 ± 0.1 ^a^	9.4 ± 0.5 ^a^
Ph 10	115.4 ± 6.9 ^a^	62.6 ± 3.3 ^a^	91.8 ± 6.3 ^a^	55.4 ± 4.2 ^a^	69.4 ± 4.3 ^a^	79.5 ± 2.0 ^a^	2.9 ± 0.1 ^a^	9.3 ± 0.2 ^a^
Ph 1-1-1	112.4 ± 8.5 ^a^	60.7 ± 2.5 ^a^	84.6 ± 5.7 ^a^	55.0 ± 5.6 ^a^	70.6 ± 5.3 ^a^	77.0 ± 2.3 ^a^	3.2 ± 0.3 ^a^	9.1 ± 0.7 ^a^
Ph 2-1-2	116.3 ± 7.4 ^a^	62.9 ± 3.3 ^a^	90.6 ± 4.5 ^a^	56.1 ± 5.5 ^a^	70.6 ± 5.0 ^a^	78.6 ± 2.4 ^a^	3.2 ± 0.3 ^a^	8.8 ± 0.6 ^a^
Ph 2-2-2	121.6 ± 7.3 ^a^	62.4 ± 3.4 ^a^	96.0 ± 4.7 ^b^	53.5 ± 5.0 ^a^	65.9 ± 4.6 ^a^	79.6 ± 2.5 ^a^	2.9 ± 0.3 ^a^	8.6 ± 0.8 ^a^
Ph 3-1-3	115.6 ± 6.8 ^a^	61.1 ± 3.5 ^a^	91.4 ± 6.2 ^a^	53.7 ± 3.5 ^a^	68.2 ± 4.1 ^a^	78.6 ± 1.8 ^a^	3.3 ± 0.3 ^a^	8.6 ± 0.6 ^a^
Ph 3-3-3	119.5 ± 6.9 ^a^	65.7 ± 4.8 ^b^	96.4 ± 5.6 ^b^	55.6 ± 4.8 ^a^	68.0 ± 4.7 ^a^	79.7 ± 2.5 ^a^	3.1 ± 0.3 ^a^	8.4 ± 0.6 ^b^
Ph 4-1-4	112.3 ± 7.7 ^a^	63.9 ± 2.8 ^a^	93.3 ± 5.7 ^a^	59.6 ± 4.5 ^a^	72.6 ± 5.0 ^a^	81.9 ± 1.4 ^a^	2.8 ± 0.1 ^a^	9.6 ± 0.3 ^a^
Ph 4-4-4	112.0 ± 7.6 ^a^	64.4 ± 3.2 ^a^	94.8 ± 3.9 ^b^	59.0 ± 4.3 ^a^	71.6 ± 4.7 ^a^	82.2 ± 1.7 ^a^	2.6 ± 0.1 ^a^	9.0 ± 0.5 ^a^
Ph 5-1-5	113.0 ± 6.8 ^a^	64.0 ± 2.0 ^a^	91.0 ± 4.1 ^a^	58.1 ± 4.0 ^a^	71.2 ± 3.8 ^a^	81.1 ± 1.9 ^a^	3.1 ± 0.3 ^a^	8.4 ± 0.6 ^b^
Ph 5-5-5	112.4 ± 7.4 ^a^	66.7 ± 2.0 ^b^	92.7 ± 4.3 ^a^	61.3 ± 4.4 ^a^	74.4 ± 4.4 ^a^	82.4 ± 1.5 ^a^	2.9 ± 0.1 ^a^	8.9 ± 0.3 ^a^
Ph 10-5-10	105.8 ± 8.3 ^a^	61.3 ± 2.5 ^a^	86.0 ± 5.4 ^a^	60.8 ± 5.4 ^a^	73.7 ± 5.8 ^a^	81.9 ± 1.7 ^a^	2.5 ± 0.1 ^a^	9.1 ± 0.3 ^a^
Ph 10-10-10	109.4 ± 12.3 ^a^	58.1 ± 5.1 ^a^	84.0 ± 8.8 ^a^	57.3 ± 6.5 ^a^	73.0 ± 6.9 ^a^	77.4 ± 3.4 ^a^	2.8 ± 0.3 ^a^	9.7 ± 0.6 ^a^
Ph 15-5-15	109.2 ± 8.9 ^a^	60.5 ± 3.4 ^a^	83.6 ± 4.9 ^a^	58.6 ± 6.2 ^a^	74.3 ± 6.1 ^a^	77.4 ± 2.8 ^a^	3.0 ± 0.2 ^a^	9.8 ± 0.4 ^a^
Ph 15-15-15	112.2 ± 9.4 ^a^	59.6 ± 6.1 ^a^	85.7 ± 5.1 ^a^	56.7 ± 7.4 ^a^	70.9 ± 7.5 ^a^	77.7 ± 3.7 ^a^	2.8 ± 0.2 ^a^	9.7 ± 0.3 ^a^

Different letters (*a*, *b*) indicate significant differences (*p* ≤ 0.05) between the control and irradiation patterns.

**Table 2:** Descriptive parameters (mean ± SEM; range) of the four sperm subpopulations (SP1, SP2, SP3, and SP4) identified in stallion fresh semen.

	SP1		SP2		SP3		SP4	
N	11,893		10,098		11,210		8142	
Parameter	Mean ± SEM	Range	Mean ± SEM	Range	Mean ± SEM	Range	Mean ± SEM	Range
VCL (µm/s)	72.7 ± 0.1	0.0–120.0	109.1 ± 0.1	71.6–181.9	120.1 ± 0.2	87.2–213.9	158.3 ± 0.3	83.1–372.2
VSL (µm/s)	45.9 ± 0.1	0.0–84.6	64.7 ± 0.2	0.9-105.6	94.5 ± 0.2	43.4–199.0	47.2 ± 0.3	0.6–221.2
VAP (µm/s)	59.9 ± 0.1	0.0–112.9	85.1 ± 0.1	34.4–129.3	112.7 ± 0.2	83.9–224.8	109.5 ± 0.2	24.1-282.5
LIN (%)	63.2 ± 0.2	0.0–100.0	60.7 ± 0.2	0.8–97.7	79.4 ± 0.1	33.8–99.3	30.0 ± 0.2	0.4–84.4
STR (%)	72.1 ± 0.2	0.0–100.0	76.6 ± 0.2	1.4–99.7	84.3 ± 0.1	35.4–100.0	43.7 ± 0.2	0.5–98.3
WOB (%)	80.6 ± 0.1	0.0–100.0	78.6 ± 0.1	28.1–100.0	93.8 ± 0.1	71.2–100.0	69.7 ± 0.2	17.6–100.0
ALH (µm)	2.2 ± 0.1	0.0–5.9	3.5 ± 0.1	0.9–6.6	2.5 ± 0.1	0.4–5.30	5.7 ± 0.1	2.0–16.9
BCF (Hz)	7.7 ± 0.1	0.0–21.0	11.0 ± 0.1	3.6–22.0	7.9 ± 0.1	0.0–20.0	8.4 ± 0.1	0.0–22.0

Data were obtained after classifying sperm cells into motile subpopulations through principal component and cluster analyses.

**Table 3:** DNA fragmentation parameters of horse sperm in control and irradiated samples. Data (mean ± SEM) are given as percentages of sperm with damaged chromatin (% DFI), mean fluorescence intensity of ssDNA (mean DFI), and percentages of high stainability (% HDS).

Patterns	% DFI	Mean DFI	% HDS
Control	15.4 ± 3.0	322.9 ± 30.1	21.1 ± 5.1
Photo 1	15.4 ± 0.8	335.5 ± 33.3	22.8 ± 8.9
Photo 2	15.4 ± 1.2	332.1 ± 19.5	22.8 ± 7.6
Photo 3	15.9 ± 2.9	327.5 ± 25.4	22.2 ± 5.1
Photo 4	14.2 ± 1.4	319.0 ± 21.3	18.1 ± 5.6
Photo 5	15.8 ± 1.4	326.9 ± 29.9	19.7 ± 8.9
Photo 10	14.6 ± 0.9	319.7 ± 25.4	19.3 ± 8.5
Photo 1-1-1	17.0 ± 1.4	326.0 ± 34.8	25.3 ± 8.1
Photo 2-1-2	15.6 ± 0.7	319.7 ± 29.0	21.1 ± 5.8
Photo 2-2-2	17.1 ± 1.7	332.6 ± 30.1	25.9 ± 6.1
Photo 3-1-3	16.4 ± 2.9	351.7 ± 46.2	22.7 ± 8.9
Photo 3-3-3	15.8 ± 2.9	330.4 ± 30.2	21.8 ± 3.1
Photo 4-1-4	14.3 ± 1.0	332.5 ± 26.4	18.7 ± 8.3
Photo 4-4-4	16.8 ± 2.9	357.7 ± 44.1	32.3 ± 7.2
Photo 5-1-5	17.5 ± 3.9	323.7 ± 28.1	28.2 ± 12.2
Photo 5-5-5	17.5 ± 2.7	350.0 ± 37.5	31.6 ± 6.0
Photo 10-5-10	18.4 ± 3.8	341.4 ± 27.0	31.9 ± 7.0
Photo 10-10-10	18.4 ± 3.3	324.6 ± 27.4	31.4 ± 10.4
Photo 15-5-15	19.3 ± 4.5	313.7 ± 24.3	29.7 ± 9.2
Photo 15-15-15	18.3 ± 4.3	314.5 ± 19.3	25.1 ± 9.6

No significant differences between control and irradiated samples were observed (*p* > 0.05). DFI: DNA fragmentation index. HDS: High DNA stainability.
